# Therapeutic Applications of Programmed Death Ligand 1 Inhibitors in Small Cell Lung Cancer

**DOI:** 10.3390/biomedicines13020401

**Published:** 2025-02-07

**Authors:** Leena Nabipur, Michael Mouawad, Vishwanath Venketaraman

**Affiliations:** College of Osteopathic Medicine of the Pacific, Western University of Health Sciences, Pomona, CA 91766, USA; leena.nabipur@westernu.edu (L.N.); michael.mouawad@westernu.edu (M.M.)

**Keywords:** small cell lung cancer (SCLC), PD-L1 inhibitors, chemoresistance, immune checkpoint inhibitors

## Abstract

**Background:** Small cell lung cancer (SCLC) is an aggressive cancer with rapid progression, limited treatment success, and high relapse rates. Chemotherapy and radiation are standard treatments but often result in chemoresistance. PD-L1 inhibitors have gained attention for their role in enhancing tumor immunity. **Methods:** This review summarizes clinical trials involving PD-L1 inhibitors, such as atezolizumab, durvalumab, pembrolizumab, and nivolumab, in SCLC treatment. Key trials include IMpower133, CASPIAN, KEYNOTE-604, and CheckMate 331, focusing on survival outcomes and treatment efficacy. **Results:** Studies such as IMpower133 and CASPIAN demonstrate improved overall survival when PD-L1 inhibitors were added to platinum-based chemotherapy. However, outcomes in trials such as KEYNOTE-604 and CheckMate 331 varied, showing the need for refined patient selection. Adverse events (AEs) associated with these treatments were also noted. PD-L1 inhibitors offer promise in SCLC treatment, but efficacy varies across trials and patient groups. Future research should focus on better patient selection and overcoming resistance mechanisms. Addressing immune-related AEs is essential for optimizing treatment strategies.

## 1. Introduction

Small cell lung cancer (SCLC) is the most aggressive form of lung cancer, comprising about 13–16% of all lung cancers. It has an unfavorable prognosis due to its high proliferative rate and early metastasis, with poor prognostic factors including decreased performance status, weight loss, increased age, male sex, and elevated lactate dehydrogenase (LDH). SCLC is also the most common cause of paraneoplastic syndrome, leading to the syndrome of inappropriate antidiuretic hormone secretion (SIADH) and Cushing’s syndrome [1,2,3,4]. The 5-year overall survival (OS) rate of SCLC is less than <7%, with a median survival of 7 months [5,6,7]. There is a strong association with smoking relative to other subtypes of lung cancer, with 93–94% of those with SCLC having a smoking history [2,8,9]. SCLC is also associated with a high mutation burden, including the inactivation of tumor suppressor genes, such as *retinoblastoma 1* (*RB1*), and frequent mutations in tumor protein 53 (TP53) [10] compared to patients with NSCLC [8]. Mutations in *RB1* and *TP53* lead to a decrease in tumor cell apoptosis, leading to growth and increased cancer cell survival [11].

The current mainstay for treatment of SCLC is chemotherapy combined with radiation therapy. The first-line treatment regimen of combining etoposide and cisplatin for SCLC has not had any major changes for over 30 years [12]. Current treatment regimens for those with limited-stage SCLC (Stages I–III) typically consist of 4–6 cycles of cisplatin and etoposide, alongside radiotherapy and prophylactic cranial irradiation in responsive individuals; however, carboplatin is seen to be frequently substituted for cisplatin [13,14]. Side effects with most chemotherapy agents consist of bone marrow suppression, nausea, vomiting, alopecia, and fatigue, with other adverse effects specific to particular agents, such as ototoxicity, nephrotoxicity, and neurotoxicity [15]. Additionally, there is a rapid chemoresistance that greatly impacts survival, with an average of 10-month survival following the first line of chemotherapy [13]. The current second-line treatment is topotecan, which is found to have similar objective response rates (ORRs), time to progression, and OS to the previously used cyclophosphamide plus doxorubicin and vincristine [16]. Topotecan’s overall response rates are typically under 25%, and the median OS ranges between 6–9 months [16]. The limitation of therapeutic options and high resistance to chemotherapy have led to a lack of improvement in clinical outcomes and survival rates in patients with SCLC [17]. This has led to the exploration of other immunotherapies that target immune checkpoints, such as PD-L1.

Programmed death ligand 1 (PD-L1; also known as CD274 and B7-H1) plays an important role in immune suppression and tumor immune evasion. Traditionally, it was discovered to be expressed in heart, placenta, lung, and skeletal muscle tissue. It was seen to regulate T-cell proliferation and IL-10 secretion [18]. Traditionally, PD-L1 has been understood as a surface-expressed immune checkpoint molecule that binds to receptor programmed death 1 (PD-1) on T cells, leading to the inhibition and apoptosis of T cells [19,20]. Recent research has expanded our understanding of PD-L1 beyond its role as a ligand for PD-1, revealing cancer cell-intrinsic PD-L1 signaling that leads to tumor growth and survival independently of PD-1 engagement. Unlike the classical extrinsic signaling pathway that directly suppresses antitumor immunity, intrinsic PD-L1 signaling affects tumor biology through intracellular mechanisms, including regulation of proliferation, metabolism, and DNA repair [21,22,23,24]. One key mechanism of cancer cell-intrinsic PD-L1 signaling is its ability to regulate tumor growth and metabolism. PD-L1 has been shown to activate the mammalian target of the rapamycin complex 1 (mTORC1) pathway, which drives tumor cell proliferation and enhances metabolic efficiency, thus promoting glucose uptake and glycolysis, which are essential for sustaining the high proliferative rate of cancer cells [25,26]. Through modulating these pathways, PD-L1 can promote tumor survival, even in the absence of immune cell interactions. Additionally, PD-L1 is implicated in the process of epithelial–mesenchymal transition (EMT). This process enhances the invasive and metastatic potential of cancer cells. EMT is characterized by the loss of epithelial markers, increased expression of mesenchymal markers, and enhanced motility, enabling tumor cells to invade surrounding tissues and establish metastases [27,28]. This is particularly evident in aggressive cancers, such as triple-negative breast cancer (TNBC), glioblastoma, and lung carcinoma [27,29,30,31,32]. These cancers show how an increased PD-L1 expression correlates with increased tumor aggressiveness.

**Figure 1:**
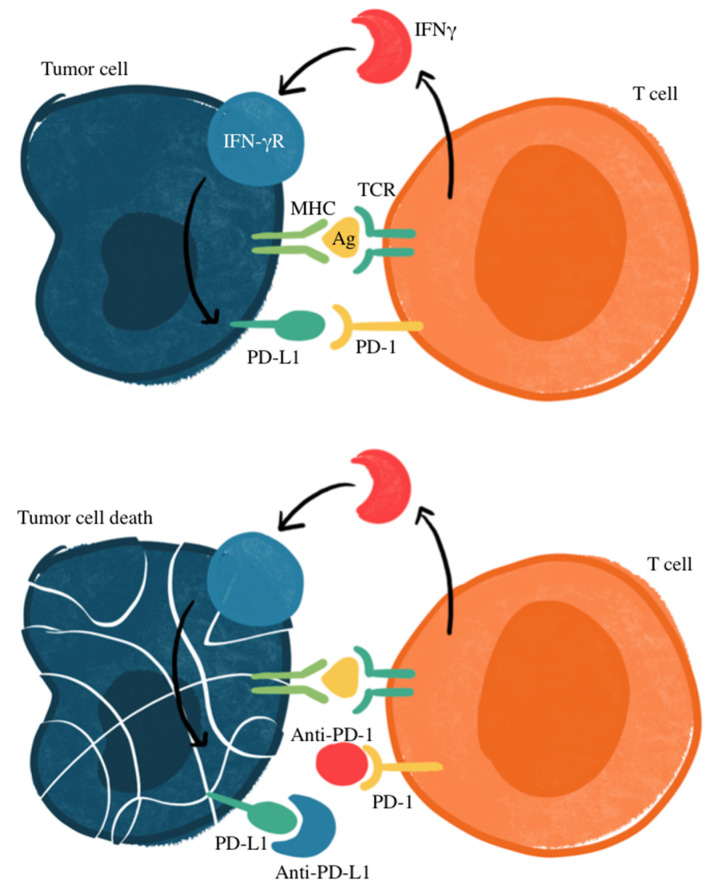
Mechanism of PD-1/PD-L1 immune checkpoint inhibition. Ag, antigen; IFNγ, interferon gamma; IFN-γR, interferon gamma receptor; MHC, major histocompatibility complex; TCR: T-cell receptor. Checkpoint proteins, such as PD-L1 on tumor cells and PD-1 on T cells, regulate immune responses by preventing excessive T-cell activation. PD-L1 binding to PD-1 inhibits T-cell-mediated tumor cell destruction (**top** panel). Immune checkpoint inhibitors targeting PD-L1 or PD-1 disrupt this interaction, restoring T-cell cytotoxicity against tumor cells (**bottom** panel).

Figure 1 illustrates the mechanism of treatment for cancers that express PD-L through antibodies that prevent PD-L1 from binding to PD-1, which prevents the suppression of T-cell activation. Research has identified how PD-L1 may be an important marker, with other studies recognizing an association of PD-L1 expression and response to targeted therapy in patients with NSCLC, making PD-L1 a potential prognostic factor for SCLC [33,34,35,36]. However, there is unavoidable heterogeneity in the prevalence of PD-L1 expression in SCLC tumor cells, thus complicating its use as a reliable biomarker. SCLC expression of PD-L1 ranges anywhere from 2% to 83%, with less expression in advanced disease states [37]. In some studies, PD-L1 has been correlated with favorable clinical outcomes for SCLC patients [38,39,40,41,42]. However, others have suggested a lack of correlation between immune checkpoint inhibition efficacy and PD-L1 status [43,44,45]. This variability includes factors within different detection methods, cut-off values used within immunohistochemistry evaluation, assessments of staining patterns, and the specificity and sensitivity of staining antibodies [46,47]. Most studies recognize a relatively low expression of PD-L1, with a higher proportion of PD-L1-positive SCLC in early stages compared to extensive-disease SCLC (ED-SCLC) [47,48,49,50,51,52]. Additionally, a higher expression of PD-L1 was seen on tumor-infiltrating immune cells rather than tumor cells [39,53,54,55].

Other checkpoint molecules are seen as potential predictive biomarkers for SCLC and actional targets to improve the response to immune checkpoint inhibitors (ICIs). A higher expression of B7-H3 has been reported to mediate pro-tumorigenic and immunosuppressive functions correlated to the reduced intra-tumoral infiltration of lymphocytes [47,56]. Furthermore, the CD47 signal that inhibits macrophage and monocyte activity was found to be highly expressed on the surface of SCLC cells [57]. Its blockage significantly enhanced the phagocytosis of SCLC cells [58].

The success of the PD-1 blockade depends on the pre-existing CD8^+^ T cells in the microenvironment that are suppressed by the PD-L1 axis. More of these T cells, as well as how close in proximity they are to the expressed PD-L1, predicts a better therapeutic response [59]. These observations suggest that evaluating the composition of the microenvironment may play a role in potential therapeutic approaches for small cell carcinomas.

Exploring the clinical outcomes and potential AEs associated with PD-L1 inhibitors, such as atezolizumab, durvalumab, pembrolizumab, and nivolumab, may provide valuable insights into the role of immunotherapy in improving outcomes for patients with SCLC.

## 2. Materials and Methods

A comprehensive review was conducted regarding the implications of PD-L1 in small cell lung cancers. The databases PubMed, Embase, and ClinicalTrials.gov were used to emphasize recent publications from 2014 to 2024, with the inclusion of additional older studies as needed to reference the established findings. The primary focus was on topics evaluating PD-L1 in clinical trials as a marker for treatment response, as well as preclinical data into pathophysiological mechanisms. The following keywords were included: “PD-L1 inhibition”, “checkpoint inhibitors”, and “small cell lung cancer”. Studies were filtered and excluded based on relevance, if they lacked detailed methodology, were editorials, or did not directly mention targeted therapies. Articles were synthesized by emphasizing studies with therapeutic efficacy, descriptive mechanisms of action, emerging therapies, and reports of clinical outcomes. Clinical trial registration is as follows: IMpower133 (NCT02763579), CASPIAN (NCT03043872), ADRIATIC (NCT03703297), KEYNOTE-604 (NCT03066778), CA209-032 (NCT01928394), CheckMate 331 (NCT02481830), and CheckMate 451 (NCT02538666).

## 3. Results

### 3.1. Clinical Trials

#### 3.1.1. IMPOWER133

The IMPOWER133 phase 1/III clinical trial was a randomized, double-blind study that compared atezolizumab (*n* = 201) in addition to a carboplatin–etoposide regimen compared to the placebo carboplatin–etoposide (*n* = 202) in those with SCLC. The median OS was 12.3 months with atezolizumab in addition to carboplatin–etoposide compared to 10.3 months with the placebo combined with carboplatin–etoposide (hazard ratio (HR): 0.76; 95% CI (confidence interval): 0.60 to 0.95; descriptive *p* = 0.0154). At the 18 months mark, 34% and 21% of patients were alive, respectively [54]. When PD-L1 expression was ≥1%, the OS was 9.7 months (95% CI: 7.6 to 17.4) in the atezolizumab plus carboplatin–etoposide cohort compared with 10.6 months (95% CI: 8.3 to 14.7) in the placebo plus carboplatin–etoposide cohort (HR: 0.87; 95% CI: 0.51 to 1.49). Furthermore, when PD-L1 expression was ≥5%, the median OS was 21.6 months (95% CI: 9.4 to not evaluable) with atezolizumab plus carboplatin–etoposide compared with 9.2 months (95% CI: 6.1 to 15.7) with placebo plus carboplatin–etoposide (HR: 0.60; 95% CI: 0.25 to 1.46) [54]. The median length of treatment was 4.7 months (range: 0–29 months) with atezolizumab and 4.1 months (range: 0–26 months) with placebo. There was a median of 7.0 doses (range: 1–39 doses) of atezolizumab and 6.0 doses (range: 1–38 doses) of placebo. Immune-related adverse effects that required treatment with systemic corticosteroids occurred in 40 patients (20.2%) in the atezolizumab plus carboplatin–etoposide group compared to only 11 patients (5.6%) in the placebo plus carboplatin–etoposide group. The most common adverse effect in either treatment group was rash (20.2% vs. 10.7%), hypothyroidism (12.6% vs. 0.5%), hepatitis (7.6% vs. 4.6%), and infusion-related reactions (5.6% vs. 5.1%). Immune-related pneumonitis occurred in five patients (2.5% vs. 2.6%) in each arm [54].

#### 3.1.2. CASPIAN

The CASPIAN phase 3 trial evaluated durvalumab and platinum/etoposide versus platinum/etoposide alone as first-line treatment for patients with ED-SCLC. This study included 537 patients who were randomized, and the results show that durvalumab plus platinum–etoposide significantly improved OS, with an HR of 0.73. The median OS was higher for the combination group at 13 months compared to 10.3 months, and 34% of patients were alive at 18 months versus 25% in the control group. The safety profiles showed similar rates of grade 3 or 4 AEs at 62%. These findings are supportive of durvalumab and platinum–etoposide as first-line therapy [60].

#### 3.1.3. ADRIATIC

The ADRIATIC phase 3 trial investigated the efficacy of adjuvant durvalumab, with or without tremelimumab, versus placebo in patients with limited-stage SCLC who had no disease progression after platinum-based chemoradiotherapy. Durvalumab significantly improved OS and progression-free survival (PFS) compared to placebo, with a median OS of 55.9 months versus 33.4 months and an HR of 0.73. Although the AEs between the two groups had similar rates, pneumonitis and immune-related events were slightly more common in patients receiving durvalumab [61].

#### 3.1.4. KEYNOTE-604

Pembrolizumab is approved as a third-line or later therapy for SCLC. The KEYNOTE-604 study was a randomized, double-blind phase III clinical trial that demonstrated antitumor activity in patients with SCLC when treated with pembrolizumab plus etoposide (*n* = 228) compared to placebo plus etoposide (*n* = 225) in individuals with untreated ED-SCLC. The results show that pembrolizumab plus etoposide significantly prolonged PFS. The estimated OS rates were 45.1% in the pembrolizumab group and 39.6% in the placebo group; additionally, the ORR was 70.6 and 61.8%. The OS, HR, and ORR favored pembrolizumab; however, there was no significant difference compared to placebo. The only group that did not have any benefit was that with baseline brain metastases [62].

#### 3.1.5. CA209-032

In the CA209-032 phase I/II trial, nivolumab was assessed as both a single agent and in combination with ipilimumab in patients with recurrent SCLC, including those who have progressed after prior platinum-based chemotherapy. The overall response rate was 15% for nivolumab alone, with six partial responses and 22.5% reaching stable disease. For the combination, the overall response rate was 25%, including one complete response and four partial responses, with 30% reaching stable disease. Several AEs included fatigue, diarrhea, and rash, with grade 3/4 events seen more frequently in the combination group. There was one fatal case of myasthenia gravis reported [63].

#### 3.1.6. CheckMate 331

In the CheckMate 331 phase III trial, they compared nivolumab monotherapy to chemotherapy of topotecan or amrubicin as a second-line treatment for patients with relapsed SCLC after initial platinum-based chemotherapy. The study found no significant difference in OS between nivolumab and chemotherapy. While PFS favored chemotherapy initially, the duration of response was longer for nivolumab at 8.3 months compared to 4.5 months for chemotherapy. Nivolumab demonstrated a more favorable safety profile. Although the exploratory analyses indicate potential benefits in patients with lower LDH and the absence of liver metastases, the PD-L1 status did not significantly correlate with improved outcomes [64].

#### 3.1.7. CheckMate 451

The CheckMate 451 phase III trial compared nivolumab combination therapy (plus ipilimumab) with nivolumab monotherapy and placebo in individuals who failed first-line platinum-based chemotherapy. The study shows that combination therapy or monotherapy did not have any significant difference in OS compared to the placebo. The OS of combination therapy was 9.2 months (95% CI: 8.2–10.2) (HR: 0.92; 95% CI: 0.75–1.12), 10.4 months (95% CI: 9.5–12.1) (HR: 0.84; 95% CI: 0.69–1.02) with nivolumab monotherapy, and 9.6 months (95% CI: 8.2–11.0) with the placebo. There were improvements in PFS in both the combination therapy group (HR: 0.71; 95% CI: 0.60–0.87) and the nivolumab monotherapy group (HR: 0.67; 95% CI: 0.56–0.81) compared to the placebo. There were no new safety concerns compared to previous reports at equal dosing and scheduling in SCLC [65].

### 3.2. Mechanisms of Resistance

There are various mechanisms of resistance to PD-L1 treatment. The first example is those who have an absence of response to the PD-L1/PD-1 blockade. This is referred to as primary resistance, and these patients never show any response to PD-1/PD-L1 targeted immunotherapy [66]. There is reason to believe this is relevant to the patient’s CD8^+^ T-cells’ inability to recognize or locate the tumor [67]. CD8^+^ T-cell recognition and T-cell activation rely on two signals: the first is the binding of a Major Histocompatibility Complex (MHC) antigen presentation to T-cell receptors, and the second is made up of co-stimulatory and co-inhibitory signals [68]. PD-1 on T cells and PD-L1 on the tumor cells or antigen-presenting cells can bind and lead to decreased T-cell activation, apoptosis, inhibition of cytokines, antigen tolerance, and even allow SCLC tumor cells to evade immune surveillance [69]. Localization defects can be due to insufficient tumor antigens for recognition. Inadequate tumor antigens may occur due to tumor tissue not being differentiated enough from their native tissue or from a low mutational burden that does not invoke a T-cell response [67]. Other mechanisms of primary resistance to PD-1/PD-L1 inhibitors include an absence of response to interferons and T-cell exclusion [66,70,71]. T-cell exclusion is a limitation of T-cell infiltration into the tumor itself due to physical barriers and immunosuppressive factors, such as inhibitory cytokines secreted by tumor cells [71].

Acquired resistance to PD-L1 inhibition is when patients initially show a response but then become unresponsive to treatment [66]. This can be due to T-cell function loss, impaired antigen presentation, or resistance to interferons that are generated by T cells [67,72,73,74,75]. Additionally, mutations affecting beta-2 microglobulin and human leukocyte antigen-A (HLA-A) can disrupt genes that impact antigen presentation, therefore reducing CD8^+^ T-cell infiltration. This diminishes the effectiveness of the PD-L1 blockade due to impaired T-cell response, regardless of PD-L1 expression [76].

**Figure 2:**
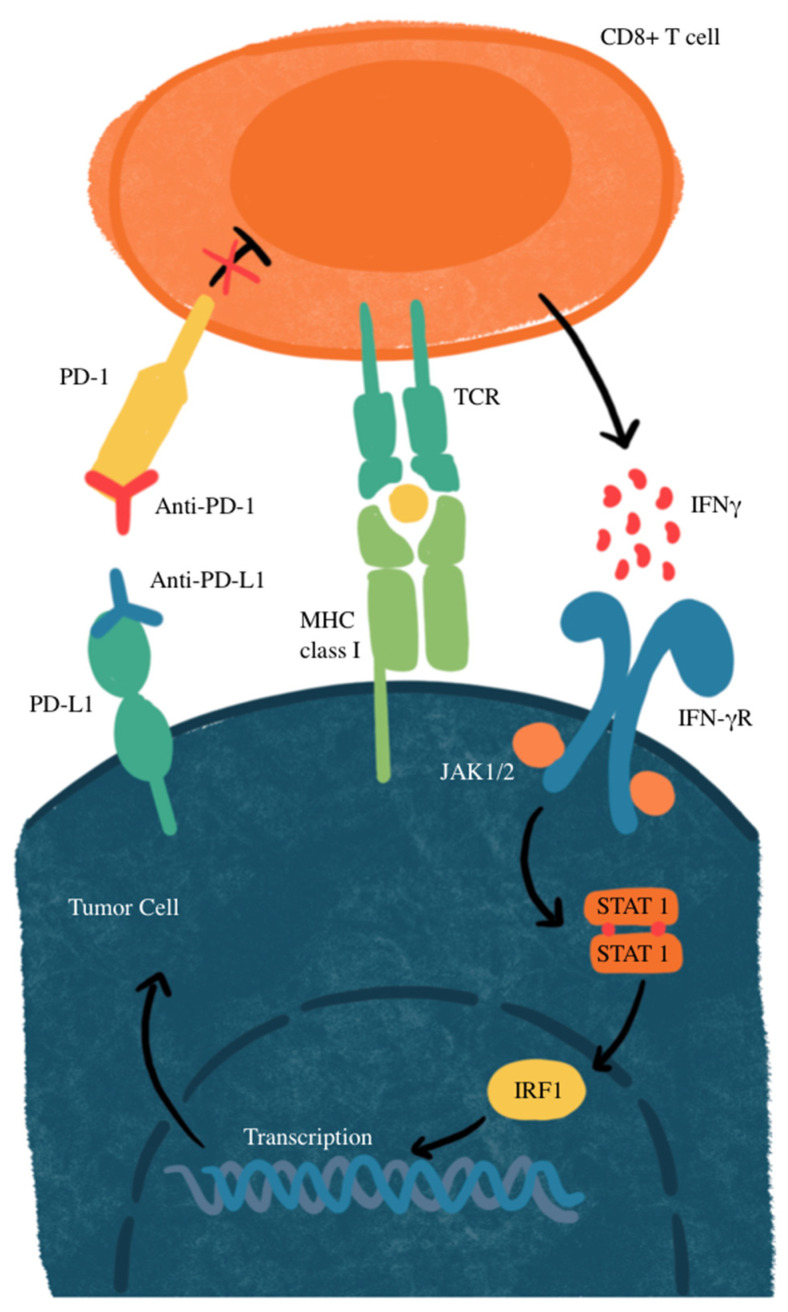
JAK-STAT-mediated PD-L1 upregulation and immune checkpoint inhibition. IFNγ, interferon gamma; IFN-γR, interferon gamma receptor; IRF1, interferon regulatory factor 1; MHC, major histocompatibility complex; JAK1/2, Janus kinase 1/2; STAT, signal transducer and activator of transcription; TCR, T-cell receptor. CD8^+^ T-cells recognize tumor antigens on MHC class I, triggering IFN-γ release, which activates the JAK-STAT pathway and upregulates PD-L1 on tumor cells. PD-L1 binds PD-1 on T cells, suppressing their activity. Anti-PD-1 or anti-PD-L1 antibodies block this interaction, restoring T-cell function and enhancing antitumor activity.

**Figure 3:**
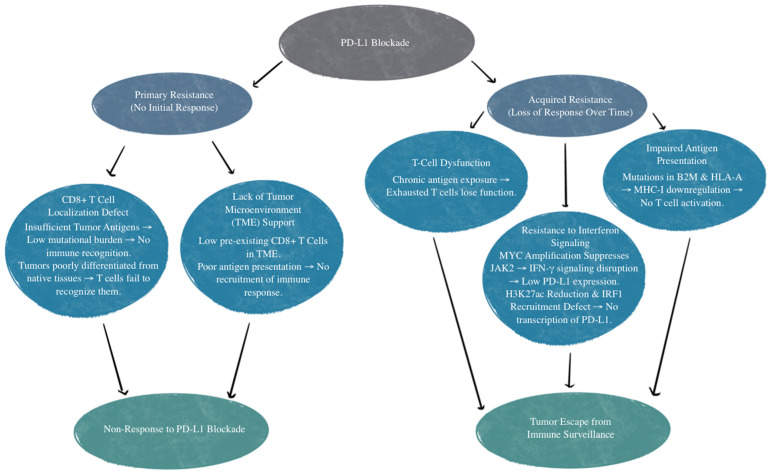
Mechanisms of primary and acquired resistance to PD-L1 blockade. TME, tumor microenvironment; B2M, beta-2 microglobulin; HLA-A, human leukocyte antigen-A; MHC-I, major histocompatibility complex class I; IFN-γ, interferon-gamma; *JAK2*, *Janus Kinase 2*; *MYC*, *MYC proto-oncogene*; H3K27ac, histone H3 lysine 27 acetylation; IRF1, interferon regulatory factor 1.

Another mechanism of resistance that SCLC has to the PD-L1 blockade is through *MYC* gene amplification. *MYC*-amplified SCLC leads to *Janus kinase 2* (*JAK2*) suppression. The mechanism of JAK-STAT-mediated PD-L1 upregulation and immune checkpoint inhibition is illustrated in Figure 2. Interferon-gamma (IFN-γ) increases PD-L1 expression through the JAK/STAT signaling pathway by promoting the expression of *CD274*, which is the gene that encodes PD-L1 [77]. Therefore, MYC amplification leads to reduced PD-L1 levels in MYC-amplified SCLC by the suppression of *JAK2*. Furthermore, even if PD-L1 is expressed in cancer cells, it is not responsive to PD-L1 due to the MYC interference of IFN-γ. This means that PD-L1 expression may not be sufficient to predict sensitivity to PD-L1-blockade immunotherapy [76]. Additionally, many neuroendocrine SCLCs that are MYC-amplified have a reduction in histone 3 acetylation at lysine 27 (H3K27ac) in the promoters of IFN-γ-stimulated genes, leading to altered chromatin accessibility and reduced transcription. This reduction leads to decreased transcription of PD-L1 and hypermethylation, in turn inhibiting the recruitment of interferon regulatory factor 1 (IRF1) to DNA. IRF1 plays an important role in activating immune response genes, including PD-L1 and other immune-related genes. In SCLCs that are partial or non-responsive to immunotherapy, there is limited IRF1 recruitment, leading to decreased PD-L1 transcription and a weakened immune response [76,78]. The mechanisms of primary and acquired resistance are illustrated in Figure 3. 

### 3.3. Advancements and Implications for Future Research and Clinical Practice

The recent literature highlights advancements in the use of PD-L1 inhibitors for first-line treatment in SCLC, particularly in combination with platinum–etoposide. Table 1 provides a summary of the clinical trials of PD-L1 inhibitors. Atezolizumab and durvalumab have shown an improvement in OS of about 2–3 months or a 10% increase, which then led to Federal Drug Administration (FDA) and European Medicines Agency (EMA) approvals for SCLC when combined with platinum–etoposide. The IMpower133 trial determined that atezolizumab extended the median OS to 12.3 months in comparison to 10.3 months in the control group with benefits regardless of PD-L1 expression. Similarly, the CASPIAN trial showed durvalumab combined with platinum–etoposide improved OS, with a 12-month median OS compared to 10.3 months in the control group. However, the recent findings indicate no statistical difference in PFS or OS among atezolizumab, durvalumab, pembrolizumab, and nivolumab when used as first-line treatments in ED-SCLC [79]. Nonetheless, all drugs showed statistical benefit over placebo, with their HRs indicating reduced risk: HR for atezolizumab: 0.77 (95% CI: 0.61–0.97); HR for durvalumab: 0.80 (95% CI: 0.66–0.97); HR for pembrolizumab: 0.73 (95% CI: 0.60–0.89); and HR for nivolumab: 0.65 (95% CI: 0.46–0.91) [79]. These findings show how valuable PD-L1 inhibitors are in extending survival in SCLC, with the inadvertent increased rate of immune-related AEs, such as rash, hypothyroidism, and hepatitis.

Emerging data also address the role of other PD-L1 inhibitors, such as pembrolizumab and nivolumab, in advanced SCLC, however, with mixed outcomes. In the KEYNOTE-604 trial, pembrolizumab improved PFS but did not significantly impact OS compared to placebo, demonstrating the need for further refinement in selection criteria, specifically in populations with brain metastases. Compared to atezolizumab in the IMpower133 trial and durvalumab in the CASPIAN trial, OS with pembrolizumab was shorter. This could be due to a multitude of reasons, but the most likely explanation for these results is decreased effectiveness or that patients during the KEYNOTE-604 trial had a greater baseline metastasis compared to the other two trials. Nivolumab, in the CA209-032, CheckMate 331, and CheckMate 451 trials showed promising findings in specific subsets of SCLC patients, especially when combined with ipilimumab, though its effect on OS was not statistically significant as a second-line therapy. Resistance mechanisms also remain a significant barrier, with primary resistance often due to a lack of CD8^+^ T-cell recognition or localization to the tumor, as well as acquired resistance from impaired antigen presentation and loss of T-cell function. These findings emphasize the complexity of PD-L1 inhibition in SCLC and how certain therapies have shown inconsistent results.

Including the PD-L1 blockade into the treatment regimen for SCLC represents a significant advance in clinical practice, especially for patients with extensive disease. Clinicians may offer therapies that can extend the median OS, a notable improvement given a malignancy that is known for its aggressive course and limited therapeutic options. PD-L1 inhibition also brings considerations of the patient selection, monitoring, and management of immune-related AEs. While these therapies have shown efficacy regardless of the PD-L1 expression levels, they require careful monitoring for adverse reactions, which may necessitate the use of corticosteroids and could impact patients’ quality of life. For patients, these agents provide the opportunity for improved survival and may serve as a foundation for further advances.

The current literature highlights several areas for future research. Although PD-L1 inhibitors, such as atezolizumab and durvalumab, have shown benefits in SCLC, their efficacy is not strongly correlated with PD-L1 expression levels. The lack of a predictive biomarker limits the ability to stratify patients based on who would be most likely to benefit from immunotherapy. Future research could focus on identifying additional biomarkers, such as tumor mutational burden, immune cell infiltration, or specific genetic mutations. Biomarker exploration may also involve analyzing IFN-γ responsiveness and other elements in the tumor microenvironment that may influence immune response and therapy resistance. In addition, the expansion of chemotherapy to include PD-L1 inhibitors has shown modest survival benefits. Research into combination therapies with other agents, such as cytotoxic T-lymphocyte-associated protein 4 (CTLA-4) inhibitors or other targeted therapies, may improve outcomes by enhancing immunity and overcoming resistance. Resistance to PD-L1 inhibitors remains an area for further understanding. Research that targets these mechanisms, such as enhancing antigen presentation, modifying the tumor environment, or combining PD-L1 inhibitors with the stimulation of T-cell infiltration, may help to address these barriers. Additional potential avenues could include gene editing technologies, oncolytic viruses, or T-cell receptor therapies. Addressing these areas for further research could lead to more tailored treatment regimens that maximize efficacy, improving the overall impact of immunotherapy in this challenging cancer type.

## 4. Conclusions

PD-L1 inhibitors have shown significant potential for improving outcomes for patients with SCLC. This is highlighted by key trials such as IMpower133 and CASPIAN, which show extended OS with atezolizumab and durvalumab when combined with platinum-based chemotherapy. These findings lay the groundwork for the management of SCLC, establishing PD-L1 inhibitors as essential for first-line treatment strategies. Mixed results from studies such as KEYNOTE-604 and CheckMate 331 indicate that the benefits of the PD-L1 blockade may vary across patient subgroups and are not solely dependent on PD-L1 expression levels. The variability in response and the challenge of resistance mechanisms underscore the need for future studies to identify reliable predictive biomarkers and optimize patient selection. Further research should also explore combination therapies and novel strategies to overcome resistance, aiming to tailor treatment approaches that maximize efficacy. Continued investigation will be crucial to fully integrate PD-L1 inhibitors into SCLC management, ensuring that therapeutic advances translate into meaningful, long-term benefits for patients.

## Figures and Tables

**Table 1:** Clinical trials evaluating PD-L1 inhibitors in small cell lung cancer.

Trial	Efficacy Highlights	Survival Rates	Adverse Effects	PD-L1 Inhibitor	Comparator
IMpower-133 (IMpower 133)	Atezolizumab improved OS to 12.3 months vs. 10.3 months in control; PFS HR: 0.77.	Median OS: 12.3 months (atezolizumab) vs. 10.3 months (control).	Immune-related AEs (rash, hypothyroidism, and hepatitis).	Atezolizumab	Placebo + CP/ET
CASPIAN	Durvalumab improved OS to 12.9 months vs. 10.5 months in control; PFS HR: 0.80.	Median OS: 12.9 months (durvalumab) vs. 10.5 months (control).	Similar immune-related AEs; manageable with corticosteroids.	Durvalumab	Platinum + ET
ADRIATIC	Durvalumab significantly improved OS and PFS in limited-stage SCLC (LS-SCLC) post-chemoradiotherapy.	Median OS: 55.9 months (durvalumab) vs. 33.4 months (placebo); HR: 0.73.	Similar AE rates between groups; pneumonitis and immune-related events slightly more common with durvalumab.	Durvalumab	Placebo after CRT
KEYNOTE-604 (KEYNOTE 604)	Pembrolizumab improved PFS but OS not statistically significant.	Median PFS: 4.5 months (pembrolizumab) vs. 4.3 months (placebo).	AEs consistent with pembrolizumab’s safety profile.	Pembrolizumab	Placebo + EP
CA 209-032	Nivolumab showed promise in certain SCLC subsets as second-line therapy.	OS not statistically significant; focus on subsets.	Treatment-related AEs higher in combination with ipilimumab.	Nivolumab	Nivolumab + ipilimumab
CheckMate 331	Nivolumab monotherapy did not improve OS over chemotherapy.	Median OS: 7.5 months (nivolumab) vs. 8.4 months (control).	Nivolumab monotherapy associated with manageable AEs.	Nivolumab	Topotecan/Amrubicin
CheckMate 451 *	Nivolumab + ipilimumab did not improve OS; slight PFS benefit.	Median OS: HR 0.92 (nivolumab + ipilimumab) vs. placebo.	Higher rates of grade 3/4 AEs with nivolumab + ipilimumab.	Nivolumab + ipilimumab	Placebo (maintenance)

CP/ET, carboplatin/etoposide; EP, etoposide/platinum; OS, overall survival; PFS, progression-free survival; ORR, objective response rate; CRT, chemoradiotherapy. Hazard ratios (HRs) represent the relative risk reduction in the treatment arm compared to control for specified outcomes. Adverse events (AEs) represent immune-related side effects commonly seen with immunotherapy agents. Grade 3/4 events indicate severe or life-threatening reactions. * Findings are specific to maintenance therapy in patients with ED-SCLC; no significant OS benefit observed in this context. Data are sourced from individual study reports, with varying population inclusion criteria, potentially impacting outcome comparisons [54,60,61,62,63,64,65].

## Data Availability

PubMed, Google Scholar, and NHI.
